# Modulation of Sleep Architecture by Whole-Body Static Magnetic Exposure: A Study Based on EEG-Based Automatic Sleep Staging

**DOI:** 10.3390/ijerph19020741

**Published:** 2022-01-10

**Authors:** Lei Yang, Haoyu Jiang, Xiaotong Ding, Zhongcai Liao, Min Wei, Juan Li, Tongning Wu, Congsheng Li, Yanwen Fang

**Affiliations:** 1 China Academy of Information and Communications Technology, Beijing 100191, China; yanglei@caict.ac.cn (L.Y.); haoyu_jiang@foxmail.com (H.J.); dingxiaotong1996@163.com (X.D.); lijuan@caict.ac.cn (J.L.); wutongning@caict.ac.cn (T.W.); 2 Zhejiang Heye Health Technology, Anji 313300, China; zhongcai.liao@heaye.com (Z.L.); min.wei@heaye.com (M.W.)

**Keywords:** static magnetic field exposure (SMFE), questionnaire, electroencephalogram (EEG), sleep staging, support vector machine (SVM)

## Abstract

A steady increase in sleep problems has been observed along with the development of society. Overnight exposure to a static magnetic field has been found to improve sleep quality; however, such studies were mainly based on subjective evaluation. Thus, the presented data cannot be used to infer sleep architecture in detail. In this study, the subjects slept on a magneto-static mattress for four nights, and self-reported scales and electroencephalogram (EEG) were used to determine the effect of static magnetic field exposure (SMFE) on sleep. Machine learning operators, i.e., decision tree and supporting vector machine, were trained and optimized with the open access sleep EEG dataset to automatically discriminate the individual sleep stages, determined experimentally. SMEF was found to decrease light sleep duration (N2%) by 3.51%, and sleep onset latency (SOL) by 15.83%, while it increased deep sleep duration (N3%) by 8.43%, compared with the sham SMFE group. Further, the overall sleep efficiency (SE) was also enhanced by SMFE. It is the first study, to the best of our knowledge, where the change in sleep architecture was explored by SMFE. Our findings will be useful in developing a non-invasive sleep-facilitating instrument.

## 1. Introduction

Sleep disturbance has been the main issue for an increasing number of individuals with the progression of society. It can lead to decreased memory and learning, gastrointestinal disorders, depression, and exacerbation of chronic conditions [1]. Approximately 30% of adults and 48% of older adults in particular experience chronic insomnia [2]. Chronic insomnia is difficult to cure using the currently available pharmacotherapy [3]. Therefore, physical therapies have been used to treat chronic insomnia [4,5]. Electric, magnetic, and electromagnetic fields have been applied to modulate sleep in a series of clinical and experimental studies [6,7,8]. Most of these treatment approaches were non-invasive and less stimulant. Therefore, the application of such therapies is promising, although the effects were not always consistent, nor a clear mechanism of action has been elaborated.

In most studies, questionnaires and self-reported scales are commonly applied to evaluate sleep quality [8]. Among them, the Pittsburgh Sleep Quality Index (PSQI) [9] and the Self-Rating Scale of Sleep (SRSS) [10] are useful tools for sleep-related psychiatric research and practice. The former measures the overall sleep quality during a period, while the latter assesses short-term sleep quality, e.g., the efficacy of a sleep disorder therapy for each night during an experiment.

Sleep has a complex architecture and includes various physiological changes that occur during the period. A person usually experiences four to six sleep cycles per night, which includes different sleep stages. The American Academy of Sleep Medicine (AASM) has divided the sleep process into five stages: awake (N0), non-rapid eye movement (N1-N3), and rapid eye movement (REM) sleep [11]. N3 is slow-wave sleep, which is the most recuperative sleep period and is often indicative of high-quality sleep [12]. Sleep staging is commonly used as an indicator in diagnosing sleep diseases and related psychiatric disorders. In contrast, self-reported questionnaires may implicitly associate with the overall sleep quality but could be undermined by subjectivity; therefore, it is difficult to discriminate the individual sleep stage by using self-reported questionnaires.

Neurophysiological analyses, e.g., using electroencephalogram (EEG), are used in the research of electromagnetic field exposure effects [13,14] and sleep quality determination [15]. Sophisticated paradigms have been developed to process sleep EEG signal into characteristics that reflect sleep rhythms, neural tension, or neural activity [16]. Sleep staging calculates the dwelling time of sleep in each stage by using time-domain signals from multiple electrodes. It traditionally requires extensive manual intervention to discern the specific EEG features. For example, the dataset for an 8-h consecutive sleep may have a volume of 500 MB if sampled at 1000 Hz. In such a case, the results are prone to human error due to fatigue [17]. Therefore, automatic sleep staging is required, and sleep staging based on the machine learning method is a promising alternative [18]. 

A great number of scientific literatures about automatic sleep staging detection were presented. The majority of these scientific literatures use single-channel EEG recordings for automatic sleep staging [19] and in most cases classified models are built on extracted features. Features are extracted from linear or nonlinear. For improving the classification accuracy and accelerating the model construction procedure, feature selection has become an important step in data preprocessing [20]. There are many feature selection algorithms, including filtering, encapsulation and embedded ones. Decision tree is a typical embedded feature selection algorithm. A decision tree by Liu et al. is suitable for sleep EEG staging due to that it could achieve feature selection for imbalanced data. Selected features are generally used as input for classic algorithms such a support vector machines (SVM), k-nearest neighbor, decision tree (DT), etc. [21]. SVM shows good generalization performance for high dimensional data due to its convex optimization problem [22].

In this study, changes in sleep architecture by static magnetic field exposure (SMFE) were evaluated. Forty-one subjects were randomly divided into two groups (real SMFE group and sham SMFE group) for participation in the experiment for four consecutive nights. Whole-body SMFE was applied by a magnetostatic mattress. During the experiment, sleep EEG was recorded, while PSQI and SRSS were used to report the individual overnight sleep quality. Twenty temporal, frequency and nonlinear metrics were extracted from the labeled sleep EEG by using the Physionet database. A decision tree (DT) was trained using data from this sleep EEG database to select a set of features for sleep staging. The acquired sleep EEG was then classified by a support vector machine (SVM). The purpose of this study is to explore whether there is an ameliorative effect of SMFE on sleep and to explore the adjuvant treatment of chronic sleep disorders.

## 2. Materials and Methods

### 2.1. Exposure System and Simulations

**Figure 1:**
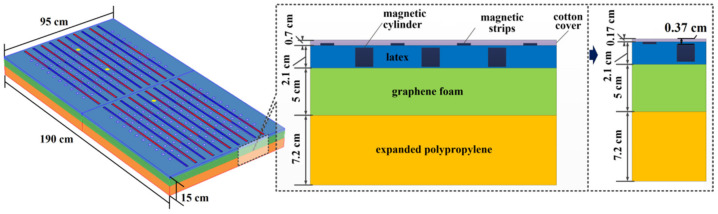
Numerical model of the magnetostatic mattress. The magnetic strips were aligned, with south poles at both ends and the north pole in the middle (S1-N-S2) or the south pole in the middle and the north poles at both ends (N1-S-N2) [24]. In the figure, red represents the south pole, and blue represents the north pole. Yellow represents the position of magnetic field measurement.

The design of the whole-body SMFE system should be compatible with the requirements of the sleep experiment. For this purpose, the exposure system was designed as a mattress (Figure 1). The dimensions of the mattress were 190.0 cm × 95.0 cm × 14.3 cm (length × width × thickness). The mattress consisted of three layers: expanded polypropylene (7.2 cm in thickness), graphene foam (5.0 cm in thickness), and latex (2.1 cm in thickness), with a layer of cotton cover (0.7 cm in thickness) outside of the mattress. The mattresses for the real SMFE group and the sham SMFE group had the same size and appearance. The magnetostatic mattress contained 220 pieces of rare earth magnetic cylinders of 600 mT (diameter: 2 cm) and 20 rare earth magnetic strips of 250 mT (length × width × thickness: 90.0 cm × 1.5 cm × 0.3 cm). The non-magnetostatic mattress contains the corresponding demagnetization cylinder and demagnetization strip by high-temperature treatment. The strips were attached to the surface of the mattress with alternate polarities. The magnetic cylinders were installed between the magnetic strips.

According to Low et al. at back posture or side posture, 90.9% to 96.1% of the human surface area was subjected to contact pressure in the range of 0 to 4.1 × 10^−^^3^ MPa. Furthermore, there was 23.11% to 29.37% human surface area in the range of 2.1 × 10^−^^3^ to 1.2 × 10^−^^2^ MPa, with average of 3.0 × 10^−^^3^ MPa, mainly at locations of head, chest and hips [23].

The gas and liquid presses system JRS-90 (JiuRong, Dongguan, China) was used to press down the mattress to simulate the deformation caused by the human body lying on the mattress. The height of the cotton cover was compressed to 0.17 cm and the relative distance between the magnetic stripe and the magnetic cylinder was shortened from 0.4 cm to 0.2 cm under 3.0 × 10^−^^3^ MPa. The distance between the magnetic cylinder and the surface of the cotton cover was 0.37 cm.

Magnetic flux density was simulated by Multiphysics v5.3a (COMSOL Inc., Stockholm, Sweden). The relative magnetic permeability of the nonmagnetic material layers was set to 1. In contrast, the magnetic objects were configured with a relative magnetic permeability of 1.05. The meshing schema used the adaptive method to discretize the finest structure.

The magnetic field strength at 0.37 cm, 5.37 cm, 10.37 cm and 20.37 cm form surface of three magnetic cylinder were measured by G93 Handheld Wide-range 3-Axis Teslameter (Coliy, Duesseldorf, Germany) for three times. As shown in Figure 1, they represent the location of the head, chest and hips respectively. Six locations were measured on the surface of each magnetic cylinder, the center of the circle and locations 0.2 cm, 0.4 cm, 0.6 cm, 0.8 cm, 1.0 cm from the center of the circle. The maximum of the six measurements was recorded as the measurement of the magnetic cylinder.

### 2.2. Subjects

Fifty male college students were recruited for the experiments. They were right-handed; had no medical or psychiatric disorders; had no alcohol, nicotine, and caffeine addiction; did not snore, and had good sleeping habits. Each subject was requested to complete the PSQI before the experiment, which was used to evaluate their recent sleep quality. The subjects were excluded from the experiments if their PSQI score was beyond 10 (i.e., below the level of good sleep quality). Based on the screening procedure, 44 subjects accepted to participate in the experiments and were randomly assigned to two groups: real SMFE group (r_SMFE) and sham SMFE group (s_SMFE). This was a single-blind experiment because that the operators were aware of the material of the mattress.

Another three subjects dropped out of the study (one from the r_SMFE and two from the s_SMFE) because they had difficulty in falling asleep due to the presence of EEG electrodes during the experiment. The r_SMFE included 21 subjects (mean age: 23.53 ± 1.94 years), and the s_SMFE had 20 subjects (mean age: 23.85 ± 1.51 years) who completed the experiment. During the daytime, the subjects were asked to maintain their normal routine activities.

### 2.3. Experimental Protocol

**Figure 2:**
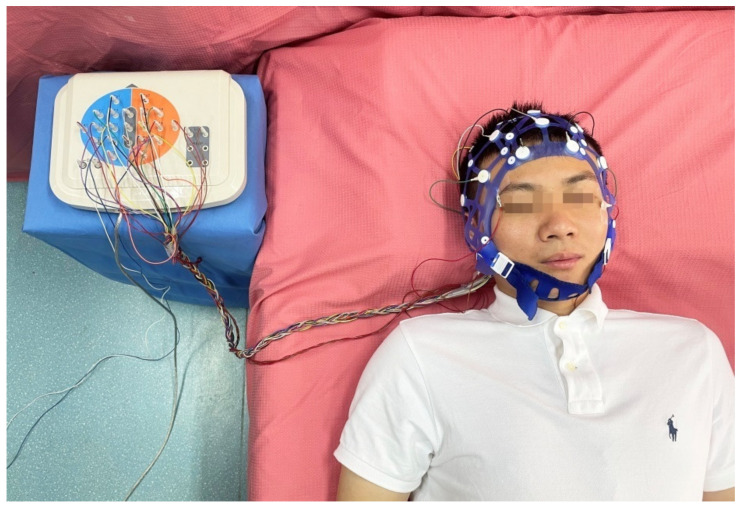
Photograph of sleep EEG recording.

The experiments were conducted in an apartment with two bedrooms, which were modified to make them fit for monitoring sleep EEG. The air conditioners were switched on to stabilize the bedroom temperature at 25 °C and to maintain normal ventilation. The ambient noise during the experiment was below 35 dB, and humidity was maintained at 50%. Bedroom windows were equipped with blackout curtains, and all light sources were switched off to ensure a dark environment [25]. The size of the bedrooms was 10 and 11 m^2^. During the sleep experiment, all subjects positioned their head toward the north direction, with their feet aligned toward the south direction. The real and sham exposure experiments were performed simultaneously in two rooms, from 10 PM to 7 AM each day. The 4 EEGs (O1, O2, C3 and C4), 2 electrooculograms (EOG: EOG1 and EOG2) are closely associated with sleep [11] and 3 EEGs (Cz, Pz and Oz) are as references and were recorded (Symtop, Beijing, China) according to the 10–20 positioning system, as shown in Figure 2. In the case of misalignment of the electrode during sleep, the missing signals from those channels were replaced with the mean signals from the adjacent channels. EEG from the other 12 channels (FP1, FP2, F3, F4, F7, F8, T3, T4, T5, T6, P3, and P4) was also measured.

### 2.4. Automatic Sleep Staging by Machine Learning

The single-channel (Pz-Oz) automatic sleep staging was classified according to the features suggested by supervised learning from the labeled open access sleep database (Physionet, https://physionet.org/content/sleep-edf/1.0.0/) (accessed on 10 October 2021). The recordings were obtained from Caucasian males and females (21~35 years old) without any medication; they contain horizontal EOG, Fpz-Cz and Pz-Oz EEG, each sampled at 100 Hz. Because the combination of the features may vary, DT was applied to identify the optimized feature set, which was subsequently used to train the SVM classifier. The trained SVM classifier was then used to identify the sleep stage based on EEG obtained from the volunteer experiment.

**Figure 3:**
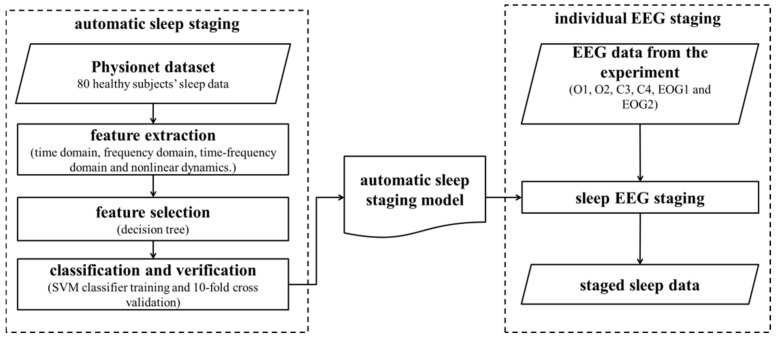
EEG-based automatic sleep staging.

A flow chart of the analysis is shown in Figure 3.

#### 2.4.1. EEG Preprocessing

EEGLAB (https://sccn.ucsd.edu/eeglab/index.php) (accessed on 15 November 2021) was used to preprocess the EEG signals. First, the EEG data were resampled to 100 Hz. The signals were further filtered with a 0.5~40 Hz band-pass filter. Because of the misalignment of the electrodes during sleep, abnormal signals were supplemented by spatial-weighted averaging from the adjacent channels. Independent component analysis identified and removed the interference components as eye movement.

#### 2.4.2. Classifier Training

1. Feature extraction

The selection of appropriate EEG features may benefit sleep staging. In the analysis, 20 features in both time and frequency domains, spanning from linear to nonlinear analysis, were applied in the study as candidates. According to AASM standards, an epoch-by-epoch (30 s) analysis was used for EEG scoring [11]. Table 1 summarizes the extracted features.

2. Feature selection by DT

Twenty features may include redundant information and could lead to the curse of dimensionality when being used for classification. DT [30], a machine learning algorithm used for classification and regression, was applied to feature selection. In this method, the Gini coefficient of individual features was calculated. The best feature was chosen according to the weighted Gini coefficient value, which was assigned as a root node for the new tree. Finally, by ranking the scores in descending order, the subset with the smallest Gini coefficient was selected as the optimal feature.

3. Individual sleep EEG classification

SVM is a nonlinear binary classifier [31]. To discriminate the sleep EEG into five stages, the variant of SVM using the one-vs-the-rest (OvR) strategy was applied. This method transforms a multicategory problem into a binary classification problem.

**Figure 4:**
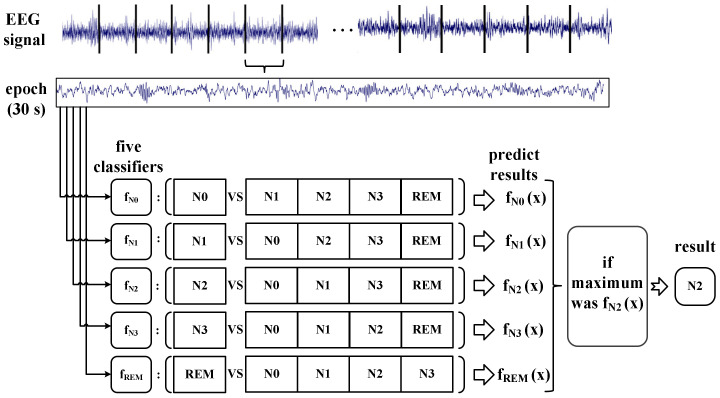
Schematic diagram of OvR. The diagram shows an example where the input epoch is classified as N2. X represents an EEG signal in an epoch.

For this study, each stage trains a binary classifier that distinguishes this stage from the other stages, implying that we created a binary classifier for each of the five stages. Thus, a total of five two-category classifiers were built: fN0, fN1, fN2, fN3, and fREM. The probability of each classifier was then estimated. The stage corresponding to the maximum of the returned estimates for all classifiers was the stage of the input epoch [32]. The schematic diagram of OvR is shown in Figure 4.

Ten-fold cross-validation was used to evaluate classification accuracy. The data were randomly divided into ten sets, with each set comprising eight subjects.

#### 2.4.3. Sleep Staging

Six sleep metrics were considered in the analysis:

Total sleep time (TST): N1_d_ + N2_d_ + N3_d_ + REM_d_ (N1_d_~N3_d_, REM_d_ represents the duration of N1~N3, REM);
N1% = N1_d_/TST × 100% (N2% and N3% are similar as N1%);
Sleep efficiency (SE): TST/TSC × 100% (TSC represents the total sleep EEG collection time, 9 h in our experiment);
Sleep onset latency (SOL): duration of switching off the lights to the beginning of first N2;
REM latency (RL): duration of the beginning of N1 to the beginning of first REM;
RL%: RL/TST × 100%.

#### 2.4.4. Verification of Staging Results

Sleep specialists were invited to visually classify the EEG of 5 subjects, which were randomly selected from the EEG data of the third and fourth nights of the two groups to verify the automatic staging results.

### 2.5. Statistical Comparison

All analyses were performed by SPSS V22.0 (IBM, Endicott, NY, USA). Sample’s *t*-test was conducted for differences in sleep parameters between the two groups (r_SMFE and s_SMFE). Paired *t*-test was used to evaluate the difference between automatic and manual staging. Bootstrap results were based on 1000 bootstrap samples. A significance level of 0.05 was used for each hypothesis.

## 3. Results

### 3.1. Simulated Magnetic Field Distribution

**Figure 5:**
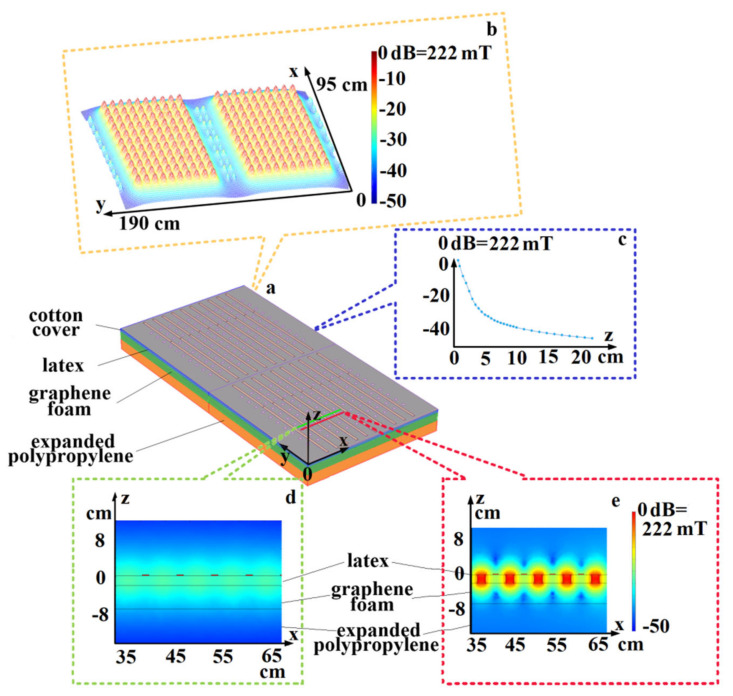
Magnetic field distribution at a distance of 0.37 cm from the surface of the magnetic cylinder with non-deformable model. (**a**): mattress model; (**b**): three-dimensional magnetic field distribution at the level of cotton cover; (**c**): decrease in magnetic flux densities above the surface of cotton cover; (**d**): magnetic field distribution of magnetic strips on the slice of y = 33 cm, (**e**): magnetic field distribution of magnetic cylinders on the slice of y = 30 cm. A coordinate system was established at the corner of the mattress, as shown in Figure 5a.

During simulation, the maximum magnetic flux intensity on the surface of the cotton cover with pressure of human was 222.2 mT, as shown in Figure 5b. The magnetic flux density decreased exponentially from the surface of the mattress, as shown in Figure 5c. The magnetic flux intensity distribution along two slices is shown in Figure 5d,e.

The measured and calculated maximum flux densities values from different location were shown in Table 2.

### 3.2. Selected EEG Features for Sleep Staging

The features were screened by DT. Eight out of twenty features were selected, namely α, MSES, FUEN, pfc, θ, SampEN, V and Kc. The resultant average classification accuracy was 91.23% on the labeled dataset (Physionet database).

**Figure 6:**
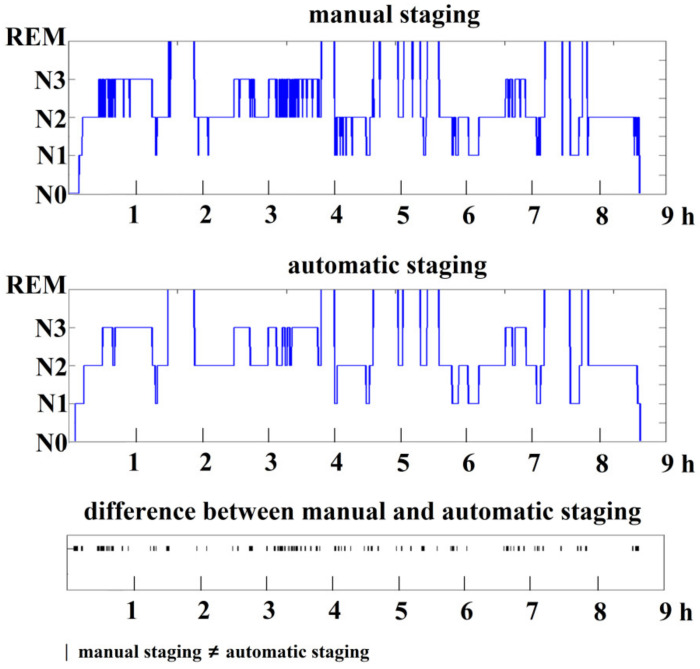
Sleep staging results obtained by manual staging and automatic staging. The horizontal axis shows sleep duration in hours.

Figure 6 shows a comparison of results obtained by manual and automatic staging for the recorded EEG in the experiments. Most of the differences between manual and automatic staging appeared at the boundaries of a specific stage.

**Figure 7:**
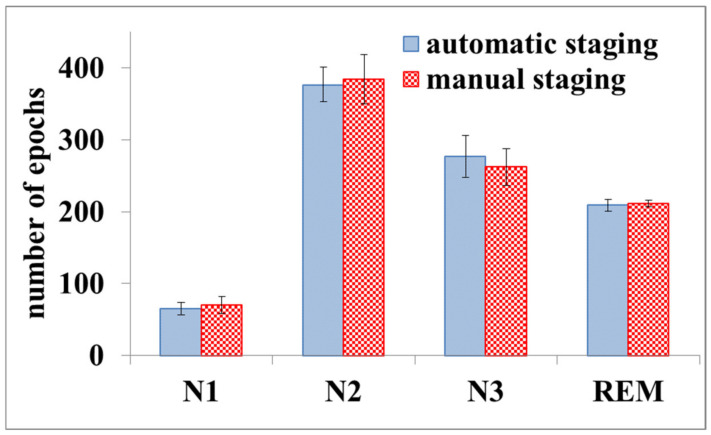
Results of automatic sleep staging and manual staging. The error bars represent the standard deviation.

Figure 7 shows a comparison between automatic staging and manual staging for the EEG obtained in this study. The worst recognition rate for N1, N2, N3, and REM wasN3, with an average value of 91.81%.

### 3.3. Changes in Sleep Quality during the Experiment

#### 3.3.1. PSQI and SRSS Rating

**Figure 8:**
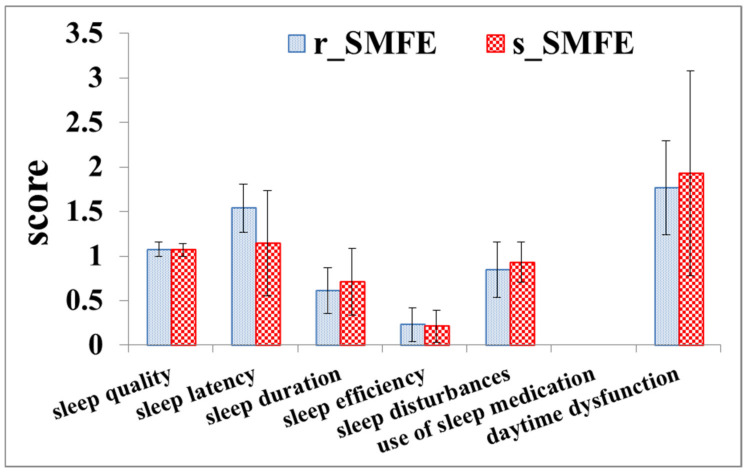
PSQI result. The error bars represent the standard deviation.

Figure 8 shows the results of PSQI when the subjects were enrolled. All the subjects had a fairly good sleep quality, with an average score of 5.99 and 6.10 in the s_SMFE and r_SMFE, respectively (0~5: very good; 6~10: fairly good; 11~15: fairly bad; 16~21: very bad). The results showed that although the subjects in the two groups had fairly good sleep quality, the sleep quality of the subjects in the s_SMFE was slightly higher than that in the r_SMFE in the month prior to the experiment.

**Figure 9:**
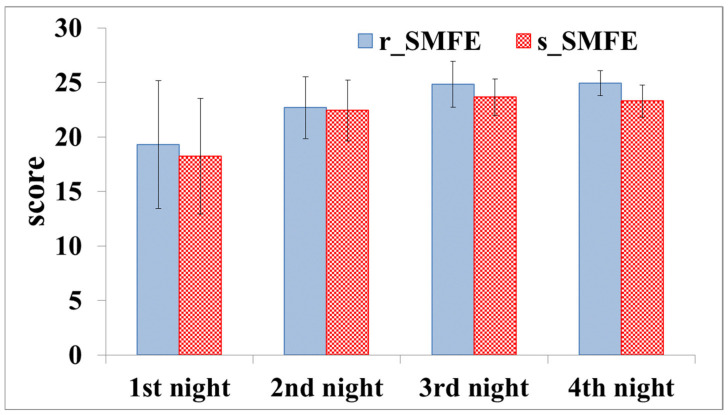
SRSS scores of four nights. The error bars represent the standard deviation.

Figure 9 shows the SRSS scores for four experimental nights. The sleep quality of all subjects on the first and second nights was not good (but in the normal range: 15~22). On the third and fourth nights, all the subjects had good sleep quality (beyond 23), and the scores of the r_SMFE were higher than those of the s_SMFE.

#### 3.3.2. Sleep Staging

N1% and N2% increased on the first two nights, while N3% and REM% decreased significantly (Table 3). The SOL on the first night exceeded 70 min, with more awake episodes (more than 1 time for each subject on average), and the RL was as long as 2.73 h. On the second night, N1% and N2% decreased, and the sleep pattern tended to be normal. On the third and fourth nights, N1% and N2% decreased continuously. N3% and REM% were prolonged, and no awakening occurred throughout the night. The sleep pattern was consistent with the normal pattern [33].

**Figure 10:**
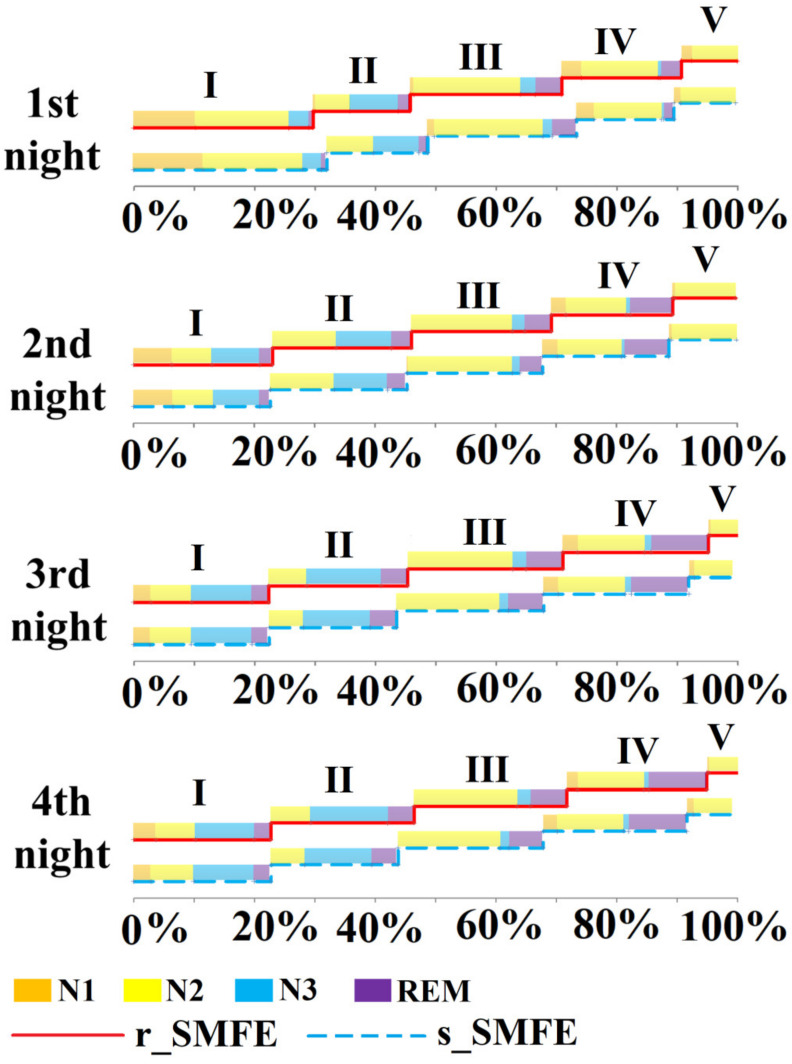
The averaged sleep cycle of the two groups during the four nights. The horizontal axis shows the total sleep duration of the night as 100%, and the graph shows the percentage of each sleep cycle; I~V represents the first cycle to the fifth cycle.

Figure 10 shows the average sleep cycle of the subjects in the different groups during the first to fourth nights. The duration of the first sleep cycle was generally longer, and the sleep cycle was irregular and incomplete. On the third and fourth nights, the r_SMFE showed longer N3 than the s_SMFE, and the completed sleep cycle was longer. This result was consistent with the subjective evaluation result. Therefore, sleep data recorded on the third and fourth nights were used as valid data for sleep quality comparison.

Table 4 shows a comparison of EEG variables among the two groups and all data follows a normal distribution. There were statistically significant differences in N2%, N3%, SE, SOL. The results showed that N2% and SOL of the r_SMFE were decreased meanwhile N3% and SE were increased in the r_SMFE.

## 4. Discussion

In this study, PSQI was used to evaluate the sleep quality of the subjects before they were enrolled in the exposure experiment. The results indicate that the subjects were at a similar level of sleep quality before the experiment. The four-night sleep quality of the subjects was evaluated using SRSS, and the detailed sleep architecture was evaluated by EEG. The results of both analyses showed that the subjects had poor sleep quality on the first night. On the one hand, the mean SRSS scores of the r_SMFE and s_SMFE were 19.31 and 18.21, respectively. On the other hand, the first RL was more than twice the normal value, indicating that the subjects had difficulty entering REM sleep, and the overall REM period accounted for approximately 10%. This may be related to factors such as discomfort caused by wearing an EEG cap and psychological stress caused by participation in sleep EEG data collection [34]. During the experiment, the subject’s adaptability to the experimental environment was strictly evaluated. If the subject reported that it was difficult to fall asleep due to EEG recording, the subject’s experiment was terminated, and all the data of this subject were abandoned (three participants were excluded from the experiment). After the first night of adaptation, the sleep quality of the remaining participants improved. In general, on the third and fourth nights, the subjects had good sleep quality (24.81 in the r_SMFE and 23.47 in the s_SMFE, in terms of mean SRSS), which was consistent with EEG results [35].

The SRSS results of the third and fourth nights indicated that the r_SMFE had higher scores than the s_SMFE, although there were no significant differences between the two groups; this suggested that the subjects in the r_SMFE subjectively felt improvement in sleep quality by the magnetostatic mattress. The results of sleep EEG in the last two nights showed significantly reduced SOL and N2%, and significantly increased SE and N3% in the r_SMFE. According to AASM, entering the N2 stage is considered as falling asleep. The reduction of SOL indicated that the subjects could fall asleep quickly. Furthermore, the increase in SE indicated that subjects had longer sleep time. The improvement for these two sleep parameters corresponded to the effect of most hypnotic treatments [36]. Unlike drug therapy, SMFE not only prolongs sleep time but also improves sleep architecture. For example, N3 was defined as deep sleep, which was found to be enhanced in the r_SMFE. During this phase, the brain organizes the memory of the daytime, and it is the key stage of the human body to restore physical strength and eliminate fatigue. This indicated that relatively short-term exposure to a weak static magnetic field could promote the deep sleep period and optimize sleep structure.

The average difference between measured and calculated with non-deformable model was 12.27% and that of deformable model was 17.69%. The difference between the measured value and the calculated value at the same measuring height is within the acceptable range. It should be noted that the location of the maximum values of the measured and calculated values are slightly different, compared to the calculated values, the location of the measured values is closer to the center of the cylindrical surface.

The magnetic flux density decreased rapidly in the body. In the present study, the magnetic flux density at the separation of 0.8 cm (epidermal and dermal layers of the human body) from the magnetic mattress was 148 mT, while it was 0.67 mT at 22 cm to the surface of the mattress (corresponding to the sagittal length of the human body because the thickness of the waist and abdomen of the human body in the 95th percentile for individuals of 18~25 years is 21.5 cm [37]. The separation between the nose tip and the back is roughly the same value [38]). The corresponding mechanism, for the effect induced by such a weak magnetic field, was difficult to be determined, but some possible explanations can be given. First, because phospholipids in cell membranes have both diamagnetic and paramagnetic properties, the lipid in the cell membrane would be realigned by the magnetic field. This interference by the magnetic field might affect various ion channels or cell structures. Therefore, the ion flux in the cell may reduce the action potential. It has been observed that a magnetic field of as low as 10 mT intensity can interact and reduce the signal traffic of the C-fiber by blocking or reducing action potential through effects on sodium flux [39,40]. Blockage of voltage-gated sodium channels reduces high-frequency repetitive firing and thereby promotes sleeping [41]. Blocking of other channels, e.g., the potassium internal rectifying channels, may also produce inhibition of the firing of neurons [33]. Study of the kinetics of oxyhaemoglobin auto-oxidation revealed decreases in the auto-oxidation reaction rate of 2~5.9% and 10~17%, under the effect of static MFs of strengths 100~250 mT and 350~400 mT, respectively [42]. Further, it has also been observed that weak magnetic fields can increase the release of oxygen from hemoglobin, thereby enhancing partial pressure of tissue oxygen and improving oxygen delivery to tissues, and thus benefiting sleep.

The abovementioned mechanisms were based on the finding at the magnetic field strength of around 10 mT, and the change in the peripheral nervous system (PNS) benefited the sleep of the subjects. Although the magnetic field strength at the brain level was 0.73 mT, which was only a magnitude higher than the strength of the earth’s magnetic field, the change in EEG by the central nervous system (CNS) exposure to such a low static magnetic field could not be ruled out. Wang et al. [43] found the alpha-band EEG discriminated in response to different geomagnetic field stimuli. The modulation of EEG of such a waveband was also detected in our experiments (as the classification feature identified by DT). The underlying mechanism was the presence of a ferromagnetic transduction element, which was directly responsive to both time-varying and static magnetic fields and was sensitive to field polarity [43]. Sensory systems generally exhibit response specificity and neural tuning to the local environment (Block, 1992), and they can be less responsive or nonresponsive to unnatural stimuli [44].

The alignment of the magnets on the mattress was designed to achieve a steep local gradient (as shown in Figure 5b) because the field gradient was thought to play an important role in the resultant biological effect [40,45], in addition to field intensity. A steep gradient field can help further reduce the action potential firing [46] as the resultant Lorentz force could lead to different velocities of the ion flux. This interferes with the action potential firing, similar to the spatially uniform but time-varying magnetic field [47].

Sleep architecture varies with sex, age, and race [48]. To evaluate the applicability of the effects on a wide population, future work should include subjects from other age groups. In this experiment, all light sources were turned off, and only the line supplying power to the sleep EEG was energized. There is a modest association between residential exposures to elevated magnetic field intensity and insomnia complaints [49]. Other the control of other electromagnetic components in the sleep laboratory would be discussed in future work (low, mainly power, frequency fields from electrical supplying system and radiofrequency fields from radio communication networks).

## 5. Conclusions

The subjective and objective analysis suggest that SMFE (varying across the body ranging at 0.5~150 mT) given by a magnetostatic mattress may improve sleep quality and the effects were also manifested in the changes in sleep architecture. Sleep stages were discerned with a set of eight EEG metrics using machine learning operators. The results of the last two nights during the experiment indicated reduced SOL, increased SE, and enhanced N3. These findings revealed that SMFE could improve sleep quality in terms of both sleep duration and sleep structure. The possible mechanisms have also been discussed. The present study may contribute to the development of instrumentation for promoting sleep improvement in individuals with sleep disorders.

## Figures and Tables

**Table 1:** Extracted features derived in an epoch.

No.	Feature	Abbreviation	Description If Needed
**1**	minimum value	MINV	/
**2**	maximum value	MAXV	/
**3**	arithmetic mean	AMV	/
**4**	median value	MNV	$\begin{array}{l} {\mathrm{MNV}(N:\mathrm{odd})=(x}_{\frac{N+1}{2}})\\ \mathrm{MNV}(N:\mathrm{even})=\frac{1}{2}(x_{\frac{N}{2}}+x_{\frac{N}{2}+1}) \end{array}$
**5**	standard deviation	SD	/
**6**	variance	V	/
**7**	skewness	S	$S=\frac{1}{N-1}{\sum_{n=1}^{N}[(\frac{x_{n}-\mathrm{AMV}}{\mathrm{SD}})}^{3}]$
**8**	kurtosis	K	$K=\frac{1}{N-1}{\sum_{n=1}^{N}[(\frac{x_{n}-\mathrm{AMV}}{\mathrm{SD}})}^{4}]$
**9**	center frequency [26]	fc	/
**10**	bandwidth	fσ	/
**11**	power spectral density of center frequency	pfc	/
**12**	gamma rhythm	γ	density at 25~40 Hz
**13**	beta rhythm	β	density at 13~25 Hz
**14**	alpha rhythm	α	density at 8~13 Hz
**15**	theta rhythm	θ	density at 4~8 Hz
**16**	delta rhythm	δ	density at 1.5~4 Hz
**17**	K complex	Kc	density at 0~1.5 Hz
**18**	fuzzy entropy	FUEN ^1^	refer to [27]
**19**	sample entropy	SampEN ^2^	refer to [28]
**20**	multiscale entropy	MSES ^3^	refer to [29]

^1^ FUEN parameters: *r* = 0.3, n = 2, *m* = 2 *SD*; ^2^ SampEN parameters: *m = 2*, *r* = 0.2 *SD*; ^3^ MSES parameters: *τ* = 11, *m* = 2, *r* = 0.15 *SD*.

**Table 2:** The measured and calculated maximum flux densities.

Distance from the Surface of the Magnetic Cylinder (cm)		0.37	5.37	10.37	20.37
calculated with Non deformable model (mT)	head	208.27	1.93	1.06	0.64
	chest	204.48	1.39	0.81	0.60
	hips	209.14	2.14	1.00	0.45
calculated with deformable model (mT)	head	222.2	2.08	1.12	0.68
	chest	219.49	1.48	0.86	0.64
	hips	222.33	2.30	1.07	0.47
Measured (mT, mean ± SD)	head	190.27 ± 4.7 × 10^−1^	1.66 ± 6.5 × 10^−3^	0.92 ± 2.6 × 10^−3^	0.56 ± 4.3 × 10^−3^
	chest	186.49 ± 4.0 × 10^−1^	1.24 ± 4.6 × 10^−3^	0.70 ± 1.8 × 10^−3^	0.52 ± 2.2 × 10^−4^
	hips	193.18 ± 4.7 × 10^−1^	1.83 ± 1.4 × 10^−3^	0.90 ± 1.2 × 10^−3^	0.51 ± 4.6 × 10^−4^

**Table 3:** Results of EEG (mean ± SD).

	1st Night		2nd Night		3rd Night		4th Night	
	r_SMFE	s_SMFE	r_SMFE	s_SMFE	r_SMFE	s_SMFE	r_SMFE	s_SMFE
N1%	16.20 ± 4.5	16.89 ± 4.5	9.50 ± 2.7	9.66 ± 2.7	5.72 ± 1.5	6.01 ± 1.3	5.82 ± 1.3	6.31 ± 1.1
N2%	59.22 ± 5.2	63.52 ± 6.8	53.96 ± 3.5	56.02 ± 3.5	46.11 ± 2.2	47.69 ± 1.7	45.93 ± 2.3	47.9 ± 1.7
N3%	14.08 ± 3.0	12.52 ± 4.0	19.82 ± 2.9	18.45 ± 3.3	25.46 ± 2.0	23.68 ± 1.2	25.45 ± 1.6	23.2 ± 1.3
REM%	10.50 ± 3.5	8.07 ± 3.9	16.73 ± 2.0	15.87 ± 1.6	22.51 ± 1.4	22.62 ± 1.4	22.81 ± 1.9	22.51 ± 1.5
TST (h) ^1^	5.58 ± 0.90	5.52 ± 0.87	7.46 ± 0.85	7.15 ± 0.85	7.96 ± 0.58	7.61 ± 0.72	8.16 ± 0.39	7.86 ± 0.67
SE ^2^ %	62.00 ± 10.0	61.31 ± 9.7	82.85 ± 9.5	79.42 ± 9.5	88.47 ± 6.4	84.56 ± 8.0	90.63 ± 4.3	87.33 ± 7.5
WN ^3^ (time)	1.00 ± 0.7	1.40 ± 0.8	0.10 ± 0.3	0.25 ± 0.4	0.00 ± 0.0	0.05 ± 0.2	0.00 ± 0.0	0.00 ± 0.0
SOL (min) ^4^	74.24 ± 16.8	75.15 ± 17.2	58.52 ± 15.4	57.89 ± 15.3	26.28 ± 5.9	28.46 ± 6.2	24.32 ± 7.6	31.65 ± 9.3
RL (h) ^5^	2.58 ± 0.8	2.73 ± 0.9	1.05 ± 0.3	1.20 ± 0.3	1.05 ± 0.3	1.03 ± 0.3	1.09 ± 0.3	1.02 ± 0.2

^1^ TST: total sleep time; ^2^ SE: sleep efficiency; ^3^ WN: awakening number; ^4^ SOL: sleep onset latency; ^5^ RL: REM latency.

**Table 4:** Comparisons of EEG sleep variables among the two groups.

	r_SMFE (%, Mean ± SD)	s_SMFE (%, Mean ± SD)	Difference (95% CI) *	*t* Value	*p* Value
N1%	5.77 ± 1.4	6.16 ± 1.2	0.0039(−0.002, 0.01)	1.37	0.174
N2%	46.12 ± 2.2	47.8 ± 1.7	0.018(0.009,0.03)	4.03	<0.001
N3%	25.46 ± 1.8	23.48 ± 1.2	−0.020(−0.03, −0.01)	−5.79	<0.001
REM%	22.76 ± 1.6	22.56 ± 1.4	−0.0019(−0.009, 0.005)	−0.58	0.566
SE%	89.55 ± 4.6	85.94 ± 6.6	−0.036(−0.06, −0.01)	−2.89	0.005
WT (time)	0.00 ± 0.0	0.03 ± 0.2	0.025(−0.02, 0.7)	1.03	0.308
SOL (min)	25.3 ± 6.8	30.06 ± 7.9	4.74(1.5, 8.0)	2.93	0.004
RL %	1.07 ± 0.3	1.03 ± 0.3	−0.043(−0.2, −0.07)	−0.75	0.453

* Difference = r_SMFE-s_SMFE. 95% CI: 95% confidence interval. Results with statistically significant differences have been highlighted in bold.

## Data Availability

The data presented in this study are available on request from the corresponding author. The data are not publicly available due to the data involved personal information of the subjects.
